# Millimeter-scale fluid-driven soft robots

**DOI:** 10.1093/nsr/nwaf413

**Published:** 2025-09-29

**Authors:** Rong Bian, Ningbin Zhang, Xinyu Yang, Jinhao Li, Dezhi Yang, Jieji Ren, Jiang Zou, Guoying Gu

**Affiliations:** State Key Laboratory of Mechanical System and Vibration, School of Mechanical Engineering, Shanghai Jiao Tong University; Shanghai 200240, China; State Key Laboratory of Mechanical System and Vibration, School of Mechanical Engineering, Shanghai Jiao Tong University; Shanghai 200240, China; State Key Laboratory of Mechanical System and Vibration, School of Mechanical Engineering, Shanghai Jiao Tong University; Shanghai 200240, China; State Key Laboratory of Mechanical System and Vibration, School of Mechanical Engineering, Shanghai Jiao Tong University; Shanghai 200240, China; State Key Laboratory of Mechanical System and Vibration, School of Mechanical Engineering, Shanghai Jiao Tong University; Shanghai 200240, China; State Key Laboratory of Mechanical System and Vibration, School of Mechanical Engineering, Shanghai Jiao Tong University; Shanghai 200240, China; State Key Laboratory of Mechanical System and Vibration, School of Mechanical Engineering, Shanghai Jiao Tong University; Shanghai 200240, China; Shanghai Key Laboratory of Intelligent Robotics, Shanghai Jiao Tong University, Shanghai 200240, China; State Key Laboratory of Mechanical System and Vibration, School of Mechanical Engineering, Shanghai Jiao Tong University; Shanghai 200240, China; Shanghai Key Laboratory of Intelligent Robotics, Shanghai Jiao Tong University, Shanghai 200240, China

**Keywords:** soft robotics, miniaturized robot, fluid dynamic, biomedical tool

## Abstract

Millimeter-scale soft robots (milli-SRs) promise significant advancements in biomedical engineering and inspection, enabling precise navigation in confined spaces. However, fabricating miniaturized fluid-driven soft robots is hindered by microscale forces. Here, we introduce a new universal design and fabrication approach (referred to as the mini bubble casting method) to create high-quality multifunctional fluid-driven milli-SRs. By injecting a bubble into pre-modified silicone liquid under high-stability conditions, we achieve submillimeter internal voids, overcoming interfacial-tension-induced instability. The modification strategy is guided by our theoretical model, which explains the influence of viscous resistance and interfacial tension on the dynamic behavior of the bubble-silicone interface. We successfully fabricate soft milli-actuators ten times smaller than existing works with low surface smoothness (${\rm Ra}=11.2$ nm). We demonstrate a milli-gripper handling delicate insects and a thrombus extractor for narrow vessels. We present a miniature steerable tip for bronchial navigation, improving safety and dexterity over traditional tools, showing the tremendous biomedical potential of these devices.

## Teaser

Mini Bubble Casting technique is developed to robustly fabricate millimeter-scale fluid-driven soft robots for biomedical applications.

## INTRODUCTION

**Figure 1.**
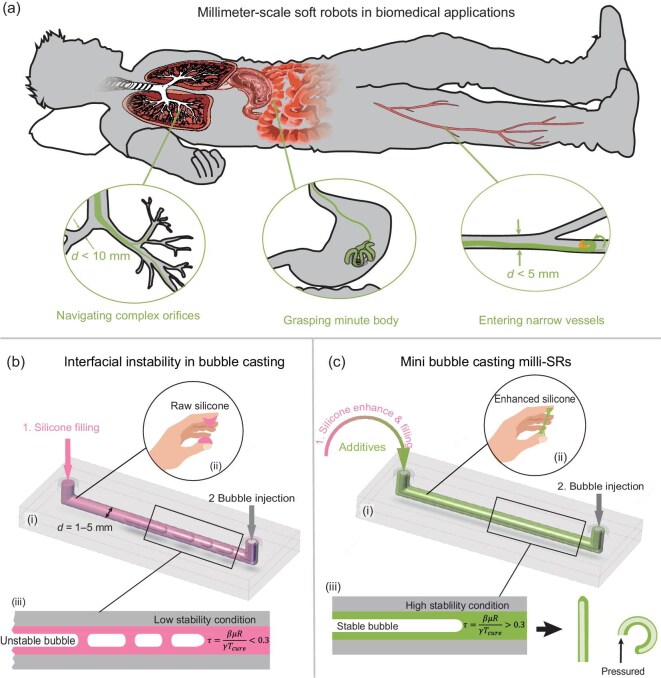
Concepts of milli-SRs and a comparison between bubble casting and mini bubble casting. (a) Milli-SRs take on biomedical tasks in confined and narrow human cavities, including navigating complex orifices, extracting foreign objects and entering narrow vessels. (b) Interfacial instability in bubble casting milli-SRs. The bubble is injected into the raw silicone liquid to form a robot void. When the robot size is scaled down to the millimeter scale, the unmodified raw commercial silicone presents a low stability condition and cannot resist the microscale forces such as surface tension, resulting in interfacial instability. (c) Mini bubble casting enables the robust fabrication of millimeter-scale soft robots by enhancing silicone stability through incorporating additives, following universal strategies guided by our theoretical model. The enhanced silicone liquid, under a high stability condition, exhibits resistance to interfacial tension and the bubble remains stable until the milli-SRs are formed.

Millimeter-scale soft robots (milli-SRs), ranging from several millimeters down to hundreds of micrometers in size, are emerging as innovative tools in biomedical engineering and tissue inspection [1–7]. These robots can navigate through organs [1,8], grip objects [9,10] and transport medical devices and drugs [11] for diagnostics and therapy, offering minimally invasive access to natural body cavities (Fig. 1a). Milli-SRs can be actuated by various mechanisms, including pressure [12,13], an electric field [14,15], a magnetic field [16–18], a chemical reaction [19,20], light [21] and ultrasound [22]. Among these, fluid-driven robots are particularly prevalent due to their ease of control and robustness [23–26]. A typical fluid-actuated soft robot comprises the silicone elastomer with one or more internal voids [27]. The pressure change in voids can be converted into deformation of soft robots. For biomedical applications, fluid-driven milli-SRs without external magnetic sources, enabling compact, lightweight medical devices [28]. Fabricated by non-metallic, MRI-compatible materials, they eliminate image artifacts and enhance safety for both clinicians and patients [29,30]. Consequently, they are well suited for complex endoluminal interventions [4], high-precision positioning [31] and environmental sensing [32] akin to magnetically driven milli-SRs.

While fluid-driven silicone robots at the centimeter scale are widely manufactured [33], fabricating their millimeter-scale counterparts remains challenging [34,35], especially in terms of robustness and stability, due to difficulties in creating smaller internal voids.

Structural defects and incompleteness frequently hinder the formation of millimeter-scale structures in traditional silicone molding methods. That is because the gravity and inertial forces, which dominate the molding process, are negligible compared to microscale forces in the low-Reynolds-number regime [35,36]. For example, in molding structures with a characteristic size of 1 mm, the Reynolds number is approximately $10^{-3}$. In this case, gravity fails to drive the silicone into the mold, and buoyancy is insufficient to lift the entrapped air. Moreover, the shapes and dimensions of robotic structures are restricted by the challenges associated with extracting long, narrow molds from the cured silicone [37]. Lately, other fabrication methods have been developed to enable novel mechanisms for mitigating failures induced by microscale forces; however, they encounter other problems. Three-dimensional (3D) printing faces limitations in material selection [38,39], and the film coating technique is also restricted to relatively simple geometries [40].

Recently, bubble casting [41] has emerged as a robust stable fabrication technique for manufacturing soft robots (Fig. 1b, i). It leverages the interfacial flow within silicone liquid to create elongated robot voids [42,43]. First, the liquid silicone is filled into a tubular mold, followed by bubble injection where the air is injected to form a long bubble in the silicone. The ideal scenario is that the bubble stabilizes under mechanical equilibrium between viscous resistance, surface tension and gravity. However, maintaining the stability of the bubble-silicone interface at the millimeter scale presents significant challenges. In experiments, we observe that the bubble fragments into a few discrete bubbles, and the silicone converges into peaks and forms bridges (Fig. 1b, iii) and Movie S1), which limits its ability to fabricate millimeter-scale robots. We hypothesize that this can be attributed to interfacial tension (Fig. 1b, ii), which increases rapidly as the scale decreases, becoming significantly greater than the viscous resistance of silicone and gravity, thereby causing an imbalance in mechanical equilibrium [44–46]. This study elucidates the mechanism of interfacial instability and its mitigation method. Whereas conventional bubble casting [41] centers on bubble forming, our focus is on the milli-bubble fragmentation and on devising a robust strategy for the reliable fabrication of fluid-driven milli-SRs.

Herein, we present a novel universal robust fabrication method, mini bubble casting (mini BC) to create fluid-driven silicone-based milli-SRs using various commercial liquid silicones (Fig. 1c), extending the lower size limit of the bubble casting process. To explore the dynamic behavior of silicone liquid at the millimeter scale, we establish a theoretical model and identify the influence of the curing time, viscous resistance and interfacial tension on the interfacial instability. Based on the model, a rheological stability condition $\tau$ is established and a universal modification strategy is proposed to enhance interfacial stability (Fig. 1c, iii) by incorporating commercially available additives. The enhanced silicone $\tau >0.3$ can withstand interfacial tension (Fig. 1c, ii), enabling theoretical minimal diameters of 0.2 mm. To illustrate its efficacy, a variety of soft actuators with different shapes and actuation modes are created, featuring dimensions one order of magnitude smaller than existing works [41,42], outer diameters ranging from 1.0 to 3.0 mm and ultra-low surface roughness (${\rm Ra}=11.2$ nm). We further demonstrate the capability of milli-SRs in interacting with delicate objects and navigating narrow natural human orifices, including a compliant and biosafe insect gripper and thrombus extractor for vascular interventional surgery. Additionally, we present a miniature flexible steerable tip with omnidirectional bending capability of up to $180^{\circ }$ that can navigate complex bronchial environments and reach the quaternary bronchus, showcasing superior safety and dexterity compared to traditional medical tools. The proposed mini bubble casting affords novel possibilities for fabricating soft robots at the millimeter scale, unlocking numerous potential applications in biomedical fields.

## RESULTS

### Interfacial instability in bubble casting

**Figure 2.**
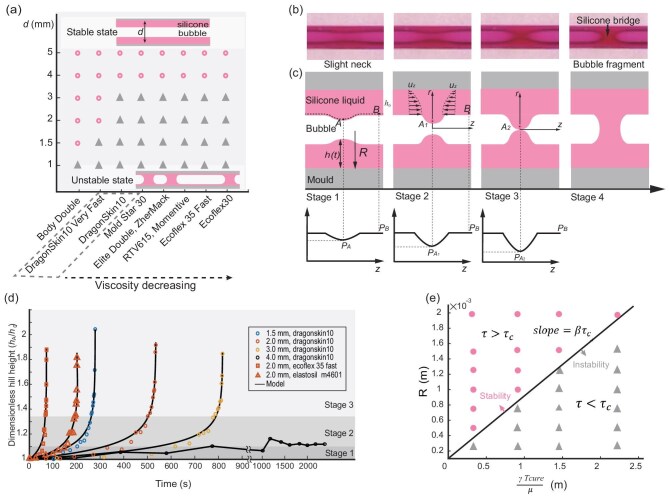
Model of interfacial instability. (a) Instability can be observed in the various commercial liquid silicones, including the VPS series used in bubble casting, as well as Ecoflex 30 and Dragon Skin 10 widely used in soft robotics laboratories. Silicones are arranged in descending viscosity; those of identical viscosity are grouped within dashed boxes. Their viscosities span 3–100 Pa s and cover the majority of commercially available silicones. (b) Four stages of interfacial instability from left to right. Diameter: 1.5 mm. (c) Diagram of silicone flow during the instability model, depicting the cross section of the silicone liquid (pink) and the bubble (white) within a tubular mold (gray). At point *A*, the formation of a silicone hill creates a pressure trap, which subsequently induces the convergence of the surrounding silicone. (d) Dimensionless hill height ${{h}_{A}}(t)/{{h}_{0}}$ versus time ($t=0$ at bubble inception) for various diameters. The silicone liquid is Dragon Skin 10 (Smooth On Inc.), Ecoflex 35 Fast (Smooth On Inc.) and Elastosil M4601 (Wacker Inc.). Solid lines correspond to (2) with fitted parameters ($\text{mean}\pm \text{SE}$): Dragon Skin 10, $n=0.0986\pm 0.005$ and $\alpha =0.12\pm 0.04$; Ecoflex 35 Fast, $n=0.112\pm 0.006$ and $\alpha =0.12\pm 0.02$; Elastosil M4601, $n=0.103\pm 0.001$ and $\alpha =0.13\pm 0.01$. Parameter explanation and the fitting method are given in the section entitled ‘Explanation of the fitting parameters’ in Supplementary data. (e) Occurrence of the interfacial instability under varying silicone conditions and within molds of different radii. Filled circles indicate stability and filled triangles indicate instability. The solid line classifies two states with the slope $\beta {{\tau }_{th}}$.

To demonstrate its broad relevance, we conduct preliminary experiments and observe interfacial instability across various commercially available liquid silicones, including the VPS series commonly used in bubble casting milli-SRs of varying diameters, as well as Ecoflex 30 and Dragon Skin 10 that are widely utilized in soft robotics laboratories (Fig. 2a).

The interfacial instability can be categorized into four distinct stages; see Fig. 2b. Initially, in stage 1, the bubble undergoes subtle unevenness near point *A*, and then it forms a notable ‘hill’ in stage 2. The silicone continues converging to point *A* and the bubble is on the verge of fracture during stage 3. Ultimately, the silicone bridges form and the long bubble fragments into several smaller, isolated bubbles in stage 4, which marks the failure of bubble casting milli-SRs.

To model the dynamic behaviors, we first consider why silicone liquid converges to point *A*. In Fig. 2c, the unevenness near point *A* creates a difference between the height of silicone layer at point *A* and point *B*, assuming that ${{h}_{A}}-{{h}_{B}}>0$. The Young–Laplace formula dictates that surface tension generates a pressure differential at point *A*, quantified as (see the section entitled ‘Young-Laplace formula’)


(1) \begin{eqnarray*}
{{P}_{A}}-{{P}_{B}}=\gamma \bigg ( \frac{1}{R-{{h}_{B}}}-\frac{1}{R-{{h}_{A}}} \bigg )<0,
\end{eqnarray*}

where $\alpha$ is the surface tension coefficient between the silicone liquid and bubble, and *R* is the mold’s inner radius. Given that ${{P}_{A}}-{{P}_{B}}<0$, the pressure gradient creates a pressure trap at point *A* that becomes deeper as ${{h}_{A}}$ increases. This places the interface in an unstable equilibrium, triggering subsequent instability at the bubble-silicone interface.

Then the silicone flow behavior can be obtained by formulating dynamic equations (see the section entitled ‘Navier-Stokes equation’) that, during stages 1–3, yield


(2) \begin{eqnarray*}
\frac{{{h}_{A}}}{{{h}_{0}}}={{\bigg ( 1-\frac{\alpha \gamma {{h}_{0}}}{2{{\pi }^{2}}{{R}^{2}}\mu }t \bigg )}^{-n}},
\end{eqnarray*}

(3) \begin{eqnarray*}
{{T}_{grow}}\approx \frac{2{{\pi }^{2}}\mu }{\alpha \gamma }\times \frac{R}{{{h}_{0}}}\times R,
\end{eqnarray*}

where ${{T}_{grow}}$ is the instability timespan, ${h}_{0}$ is the initial height of the silicone layer at point *A*, $\mu$ is the silicone viscosity. This progression stops when ${{h}_{A}}=R$ at stage 4, where the hill’s height reaches its maximum, breaking up the bubble. Figure 2d shows the growth of the dimensionless hill height ${h_A/h_o}$ over time for various diameters and silicone types, demonstrating close agreement with the theoretical Equation (2).

According to the model results, the interfacial instability is highly dependent on size and the rheological properties of the silicone liquid. The instability timespan ${{T}_{grow}}$ is positively correlated with scale *R*. Furthermore, the growth velocity of height ${{h}_{A}}$ correlates positively with the surface tension coefficient $\gamma$, and inversely with viscosity $\mu$. This explains the antagonistic effects of surface tension (promoting instability) and viscous forces (inhibiting instability).

### Instability condition and modification strategy

Determining whether instability occurs is another important aspect. To establish the instability condition, thought experiments are needed. It is noted that silicone liquid undergoes a viscosity increase and solidification during instability. Extreme stability happens when the silicone curing time ${{T}_{cure}}$ is significantly shorter than the instability timespan ${{T}_{grow}}$, causing the silicone to instantly freeze after bubble creation. Conversely, another scenario of extreme instability is where ${{T}_{grow}}$ is so small that the instability occurs rapidly in the uncured silicone. Therefore, the stability condition, denoted $\tau$, can be established as


(4) \begin{eqnarray*}
\tau =\frac{{{T}_{grow}}}{{{T}_{cure}}}=\frac{\beta \mu R}{\gamma {{T}_{cure}}},
\end{eqnarray*}

where $\beta =({2{{\pi }^{2}}}/{\alpha } )\times ({R}/{{{h}_{0}}})\approx 329$. A large $\tau$ indicates high stability, while a small $\tau$ corresponds to significant instability. Experiments validate the stability condition (Fig. 2e) and the threshold criterion can be calculated as ${{\tau }_{th}}=0.3$ (see the subsection entitled ‘Stability condition’ in the Methods section below). The fabrication yield of mini bubble casting is quantified across various instability conditions and diameters (${\rm radius}=0.5$–2.5 mm) to show the scalability of the proposed fabrication method. The improved fabrication yields confirm the scalability of the modification strategy (Fig. S3).

Based on the stability condition, we can increase the viscosity $\mu$ and decrease the curing time ${{T}_{cure}}$ until $\tau >{{\tau }_{th}}$ to mitigate interfacial instability. Notably, considering that the excessively high viscosity and rapid curing of silicone are detrimental to the operational process, the upper limitation of viscosity, $\mu _{max}$, is 100 Pa s and the low limitation of the curing time, ${{T}_{cure, min}}$, is 10 min, which means that the theoretical minimal diameter $d_{min}=0.2$ mm (see the section entitled ‘Theoretical minimal diameter by mini-BC’).

**Figure 3.**
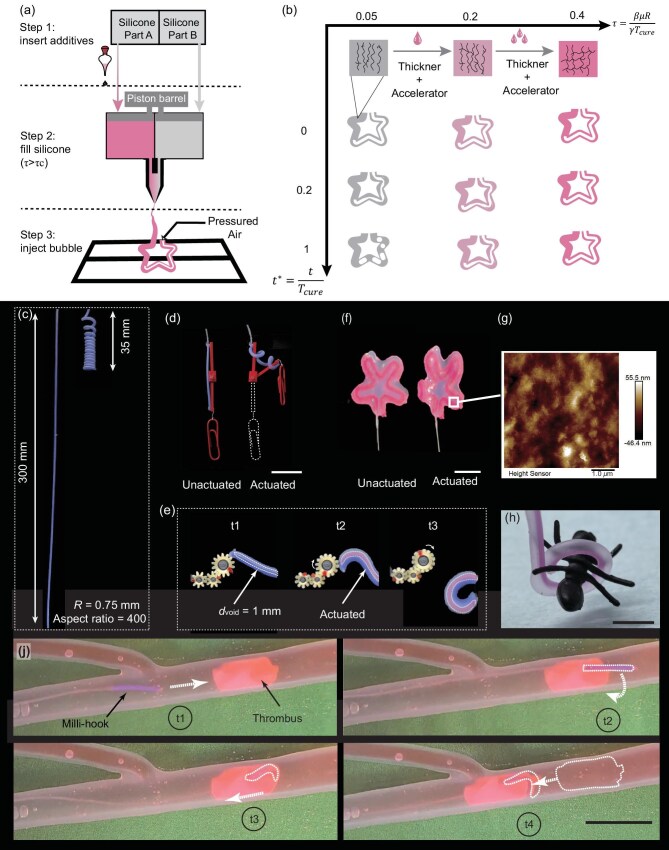
Silicone modification and milli-SRs by mini BC. (a) Silicone modification strategy. (b) The interfacial behaviors as a function of the dimensionless time $t/{{T}_{cure}}$ when creating a starlike soft actuator with a diameter of 2 mm. From left to right, we add additives to modify its stability condition $\tau$. (c–e) Soft actuators similar in shape but smaller compared to those produced by bubble casting. (c) Contractile coiling of an actuator with a radius of $R=0.75$ mm and $L=300$ mm. (d) Muscle-like contraction of a soft actuator that drives a microscale rotating pair. Scale bar: 5 mm. (e) Series images of bending actuator driving the milli-gear set. (f) A star-like actuator is attached to a thin membrane. Scale bar: 5 mm. (g) The atomic force microscope image of the void surface of the soft actuator. (h) A milli-gripper grasping and holding an ant model. Scale bar: 5 mm. (j) A milli-gripper with a bendable tip enters the vessel (inner diameter of 8 mm) and withdraws a thrombus simulator. Its contour is delineated with a dashed line for enhanced clarity. Scale bar: 10 mm.

Figure 3a shows the modification strategy. Firstly, the commercial additives, thickeners and accelerators are inserted into part A of silicone before both components are canned to injection cartridges. Taking the fabrication of 1.5-mm soft actuators with Dragon Skin 10 as an example, the unmodified silicone has a condition of ${{\tau }_{0}}\approx 0.05$. Based on sheets from suppliers, we insert the thickener (Thi-Vex, Smooth-On Inc., 3% of part A) and the accelerator (Plat-Cat, Smooth-On Inc., 1% of part A in weight) into silicone A. The new condition in the mixed silicone liquid is $\tau \approx 0.4$. Then, the modified silicone is mixed in the mixing nozzle and injected into the mold. After a waiting period, the air is injected from another side and forms a long bubble in the silicone liquid. Figure 3b shows the interfacial behaviors under different conditions. Silicone with high $\tau$ shows interfacial stability. The process is shown in Movie S2. For other commercial silicone, the suggested additives and their dosage are listed in Table S1. Excluding silicone curing, total fabrication requires 10 min; including curing, the process spans 20–70 min (Table S2).

### Milli-SRs by mini bubble casting

We successfully fabricated various milli-SRs that are similar but an order of magnitude smaller than those made by bubble casting. To demonstrate the stability of mini BC, a contractile coiling soft actuator ($R=0.75$ mm, $L=300$ mm) with aspect ratio $L/R$ as high as 400 is created in Fig. 3c and Movie S3 (its counterpart $R=5$ mm in bubble casting). Its contractile ratio of original length to minimal length can reach up to 8.5 times and it generates a contractile force of $\approx$400 mN (Fig. S8). This millimeter-scale actuator can serve as a driver for micro-mechanical systems, analogous to the role of an electric motor in large-scale machinery. The contractile milli-actuator drives a microscale rotating pair lifting a minuscule paper clip (Fig. 3d and Movie S3). The bending milli-actuator spins a milli-gear set (Fig. 3e and Movie S4). We also create similar but smaller five-point star-shaped anisotropic actuators (Fig. 3f and Movie S5) and cross-like actuators (Fig. S2 and Movie S6). They are 10 mm in diameter and one order of magnitude smaller than their bubble-casting counterpart in size (80 mm in diameter).

The surface roughness of milli-SRs is measured at ${\rm Ra}=11.2$ nm using an atomic force microscope (MFP-3D, Oxford Instruments) equipped with a stylus tip radius of 1.0 $\mu$m, as shown in Fig. 3g. The roughness is several orders of magnitude smaller than its counterpart fabricated by casting (${\rm Ra}=1$–$10\ \mu$m [47]) and 3D printing (${\rm Ra}=6\ \mu$m [38]). We quantitatively evaluate the wall-thickness uniformity (Fig. S4); details to ensure consistency are provided in the section entitled ‘The uniformity of the robot’s wall thickness’. The milli-SRs withstand a thousand loading cycles without any visible degradation in deformation performance (see the section entitled ‘Motion repeatability’ and Fig. S6). Mini bubble casting is benchmarked against alternative fabrication routes in the section entitled ‘Comparative benchmarking of millimeter-scale fabrication techniques’.

Because of their delicacy and inherent safety, the milli-actuators are proficient in grasping tiny objects and entering narrow tubes. A soft bending actuator with a diameter of 1.5 mm can function as a milli-gripper of microscale objects that are easily deformed and damaged by rigid grippers. The ant model is gripped and held without damaging its body (Fig. 3g). Dislodgement of the ant from the milli-gripper requires a pulling force of $\approx$220 mN (Fig. S8). We can also create a soft milli-hook whose tip easily bends and body hardly deforms under a single pressure by programming the mini-BC process (Fig. S7). The hook penetrates the narrow canal, engages the foreign object and withdraws it intact (Fig. 3j; Movie S7). Free from external constraints, the deflected tip forms a precise circular arc (Fig. S5); its curvature–pressure relationship is quantified in the section entitled ‘Relationship between pressure and curvature’). All milli-SRs shown in Fig. 3 are pneumatically actuated via compressed air supplied by a precision syringe pump (Fig. S10).

### Biomedical application

**Figure 4.**
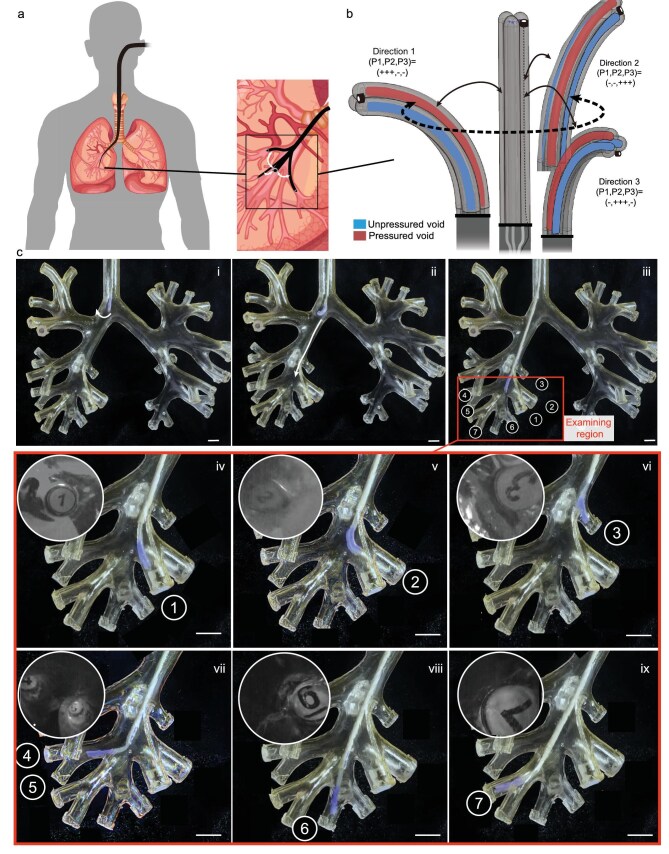
Millimeter-scale soft bronchoscope. (a) Overview of soft bronchoscope steering in the bronchus. (b) The steerable tip has two bending degrees of freedom actuated by three inner pressured voids and the micro-camera captures images from various directions. (c) The soft bronchoscope steers from the trachea (i–ii) to six fourth-order bronchi (iii–ix) and the micro-camera detects the numbered markers visible in the endoscopic view shown in the top-left corner of each subfigure. Scale bars: 5 mm.

Bronchoscopy is a common procedure for examining lung diseases, using a slender bronchoscope (2.2–6.3 mm) with a micro-camera for clinical evaluation. Its steerable tip, controlled by multiple cables, allows precise navigation through the bronchial tree (Fig. 4a). However, commercial bronchoscopy can cause complications like tissue damage and bleeding. To mitigate these risks, the use of soft materials presents an inherently safer alternative, reducing pain and promoting faster recovery.

Here, we designed an omni-curved bronchoscope tip composed of milli-actuators capable of reaching the quaternary bronchus (Fig. 4b). The tip incorporates three 1.5-mm soft actuators fabricated by mini BC with Dragon Skin 10 (Smooth-On Inc.) and a micro-camera (TA10, 720p, $0.65\times 0.65$ mm$^2$) equipped with two miniature white LEDs. The actuators are arranged in parallel and glued together, with the camera fixed at the tip. Selective pressurization of individual chambers produces localized elongation, inducing deflection of the tip (Fig. S9). For enhanced safety, normal saline is used as the pressurizing fluid in case of accidental explosion. The soft material, with a Young’s modulus of less than 150 kPa and demonstrated biocompatibility, minimizes the risk of tissue damage, ensuring that the tip is gentle enough to navigate the bronchial walls without causing harm. It is dexterous with a maximum bending angle of $180^{\circ }$ (Fig. S4d) that can meet the need for navigating the bronchus.

To evaluate its potential for biomedical applications, we assess its capabilities to steer and detect in a realistic setting. A real-size, resin bronchus model, which accurately mimics the human bronchial environment, is used for this purpose. The model is transparent to allow for presentation (Fig. 4c). The task of the soft bronchoscope is to insert into the main bronchi ($d=15$–17 mm; Fig. 4c, i), bend to the right into the right lobar bronchi ($d=4$–10 mm; Fig. 4c, ii) and reach the right lower lobar bronchus to examine the subsegmental bronchus ($d=2$–4 mm; Fig. 4c, iii). A number is added at the end of each subsegmental bronchus, and a special marker ‘$+$’ is glued to the bronchus labeled 1.

The path from the main bronchi to the right lower lobar bronchus spans diameters ranging from 17 to 4 mm, over a distance of 25 cm. In Fig. 4c, iv–ix, we experimentally demonstrate that our soft bronchoscope can complete this task (Movie S8). The operator holds the soft bronchoscope and manually manipulates the switch to adjust the inner pressure of the milli-actuators. The operator can control the bending of the soft bronchoscope in the desired direction. The micro-camera on the top of the soft bronchoscope detects the marker ‘$+$’ near the branch labeled 1 in Fig. 4c, iv as well as labels 2–7 in Fig. 4c, v–ix.

## DISCUSSION

This work elucidates and suppresses the interfacial instability in bubble casting milli-SRs, thereby pushing the attainable lower bound of the technique to the millimeter regime. Our study reveals that interfacial tension and viscous resistance act as antagonistic regulators of interfacial instability, a coupling previously unreported [37,41,42]. A dimensionless stability condition $\tau$ is introduced to predict the onset of instability; guided by it, we further prescribe modification strategies that avert the unstable behavior. Whereas conventional bubble casting focuses on bubble generation, our work centers on instability theory and mitigating bubble instability.

The mini-BC technique explains and eliminates the interfacial instability in bubble casting milli-SRs, thereby broadening the applicability of bubble casting at the millimeter scale. Our study reveals that interfacial tension and viscous resistance generally exert antagonistic effects on interfacial instability when a milli-bubble forms in the silicone liquid, a phenomenon not previously addressed in the literature [37,41,42]. Specifically, we propose the critical stable condition $\tau$ to predict the occurrence of interfacial instability and modification strategies to avoid the interfacial instability. This approach facilitates the fabrication of shape-variable soft robots with dimensions an order of magnitude smaller than those previously achieved through bubble casting. We show the utility of milli-SRs in driving micro-mechanics, grasping tiny objects and entering narrow tubes. Notably, we present a significant case of a soft bronchoscope that can navigate complex bronchial structures and perform detecting tasks, underscoring the great potential of mini-BC milli-SRs in the biomedical field.

Given the versatility of mini BC, we aim to extend its applications across other domains within biomedicine in the future. Coupling milli-SRs with novel materials can extend their capabilities beyond single-elastomer designs, such as shape-memory alloys for tunable stiffness and hydrogel coatings for targeted drug elution. Furthermore, integrating *in situ* sensing modules—capable of real-time quantification of temperature, pressure and chemical composition—into milli-SRs constitutes a pivotal strategy for advancing next-generation process intensification. In summary, our mini BC provides a new approach to the design and fabrication of milli-SRs, offering innovative solutions to interfacial instability at the millimeter scale.

## METHOD

### Stability condition

The stability condition $\tau >{{\tau }_{the}}$ can be reformulated as


(5) \begin{eqnarray*}
R>\frac{{{\tau }_{th}}}{\beta }\frac{\gamma {{T}_{cure}}}{\mu } \quad \rm {(stable\,\,state)},
\end{eqnarray*}

(6) \begin{eqnarray*}
R<\frac{{{\tau }_{th}}}{\beta }\frac{\gamma {{T}_{cure}}}{\mu } \quad \rm {(unstable\,\,state)}.
\end{eqnarray*}

We mark the stable state with pink and the unstable state with gray in Fig. 2e. A linear classifier can separate them with a slope ${{{\tau }_{th}}}/{\beta }=9.1\times {{10}^{-4}}$. Therefore, the threshold criterion can be calculated as ${{\tau }_{th}}=0.3$.

### Silicone modification

We modify the dual-component platinum cure liquid silicone that is most commonly used in soft robotics labs. To enhance the class of liquid silicone, such as Dragon Skin 10, Platinum Silicone Cure Accelerator (Plat-Cat) and Thixotropic Agent (Thi-Vex) are chosen. For other kinds of commercial silicone, the corresponding accelerator and thickener can be obtained from silicone suppliers. Additives (Plat-Cat and Thi-Vex) are mixed into part A of Dragon Skin 10 at weight ratios of 1:20 and 1:100, respectively, using a glass stirring rod. The mixture is then transferred to the left chamber of the cartridge. Subsequently, part B is poured into another cartridge part, and the silicone cartridge undergoes defoaming in a vacuum chamber ($-95$ kPa, 20 min). The evacuation rate should be no more than 10 Pa/s in case of liquid spillage. Following defoaming, we seal the modified silicone into a cartridge with pistons. To mitigate the adverse effects of phase separation in silicone, the sealed cartridge should be stored in a horizontal position to maintain component homogeneity during extrusion. Storage should be at room temperature (73 $^{\circ }$F or 23 $^{\circ }$C) away from direct sunlight. To preserve rheological stability, the material should be used within two weeks.

### Injection system

The injection system includes a two-component cartridge gun (MPD50, COX Inc.), cartridges ($50 + 50$ ml, Runze Inc.) and mixing nozzles (F1 XL3.2-16S, Runze Inc.). After installing the mixing nozzle on the cartridge, the cartridge is placed into the gun. Once the gun is triggered, 400 kPa of pressure is applied to extrude both silicone parts into the mixing nozzle in a common weight of 1:1. The initial portion of silicone extruded is recommended to be discarded to avoid issues arising from non-uniform ratios. We connect the nozzle exit and the mold with an elastic silicone tube.

### Rheological protocols

The viscosity of the silicone liquid is characterized by a rotational rheometer (Discovery Hybrid rheometer, TA Instruments Inc.) equipped with a parallel plate. The sample is freshly extruded silicone from the injection system that is thoroughly mixed prior to sampling. The surface tension of the silicone was determined using the drop weight method, with measurements conducted on OCA25 (Dataphysics Instruments Inc.).

### Fabrication of the milli-hooker

We fabricate the milli-hooker with silicone (Dragon Skin 10, Smooth on Inc.) in a tubular mold with a diameter of 1.5 mm and a length of 100 mm. The silicone is modified by inserting thickener and accelerator. We propel the bubble in the liquid silicone to the waiting point M and segment #1 forms and rises under buoyancy (Fig. S7a). After a waiting time, we propel the bubble to the outlet to form segment #2 of the bubble. The silicone molecules have undergone cross-linking, preventing segment #2 from rising (Fig. S7b). Upon curing, the axially variable void enables the milli-actuator to deform into a hook-like shape (Fig. S7c).

## Supplementary Material

nwaf413_Supplemental_Files

## References

[1] Kim Y, Parada GA, Liu S et al. Ferromagnetic soft continuum robots. Sci Robot 2019; 4: eaax7329. 10.1126/scirobotics.aax7329 33137788

[2] Yan Y, Wang T, Zhang R et al. Magnetically assisted soft milli-tools for occluded lumen morphology detection. Sci Adv 2023; 9: eadi3979. 10.1126/sciadv.adi3979 37585531 PMC10431716

[3] Thai MT, Phan PT, Tran HA et al. Advanced soft robotic system for in situ 3D bioprinting and endoscopic surgery. Adv Sci 2023; 10: e2205656. 10.1002/advs.202205656 PMC10131836 36808494

[4] Kim Y, Genevriere E, Harker P et al. Telerobotic neurovascular interventions with magnetic manipulation. Sci Robot 2022; 7: eabg9907. 10.1126/scirobotics.abg9907 35417201 PMC9254892

[5] Hu W, Lum GZ, Mastrangeli M et al. Small-scale soft-bodied robot with multimodal locomotion. Nature 2018; 554: 81–5. 10.1038/nature25443 29364873

[6] Wu Y, Dong X, Kim J et al. Wireless soft millirobots for climbing three-dimensional surfaces in confined spaces. Sci Adv 2022; 8: eabn3431. 10.1126/sciadv.abn3431 35622917 PMC9140972

[7] Zhang Y, Yang D, Yan P et al. Inchworm inspired multimodal soft robots with crawling, climbing, and transitioning locomotion. IEEE Trans Robot 2022; 38: 1806–19. 10.1109/TRO.2021.3115257

[8] Van Lewen D, Janke T, Lee H et al. A millimeter-scale soft robot for tissue biopsy procedures. Adv Intell Syst 2023; 5: 2200326. 10.1002/aisy.202200326 37637939 PMC10456987

[9] Roh Y, Kim M, Won SM et al. Vital signal sensing and manipulation of a microscale organ with a multifunctional soft gripper. Sci Robot 2021; 6: eabi6774. 10.1126/scirobotics.abi6774 34644158

[10] Bian R, Zhang N, Yang X et al. A variable stiffness soft actuator with a center skeleton and pin-socket jamming layers. In: Liu H, Yin Z, Liu L et al. (eds.) Intelligent Robotics and Applications. ICIRA 2022. Cham: Springer, 2022, 325–32.

[11] Yang Z, Xu C, Lee JX et al. Magnetic miniature soft robot with reprogrammable drug-dispensing functionalities: toward advanced targeted combination therapy. Adv Mater 2024; 36: 2408750. 10.1002/adma.202408750 39246210

[12] Phan PT, Thai MT, Hoang TT et al. Hfam: Soft hydraulic filament artificial muscles for flexible robotic applications. IEEE Access 2020; 8: 226637–52. 10.1109/ACCESS.2020.3046163

[13] Li Y, Peine J, Mencattelli M et al. A soft robotic balloon endoscope for airway procedures. Soft Robot 2022; 9: 1014–29. 10.1089/soro.2020.0161 34813373 PMC9595649

[14] Diwakar N Kunti,Miloh T et al. AC electrohydrodynamic propulsion and rotation of active particles of engineered shape and asymmetry. Curr Opin Colloid Interface Sci 2022; 59: 101586. 10.1016/j.cocis.2022.101586

[15] Lee S, Moghani M, Li A et al. A small steerable tip based on dielectric elastomer actuators. IEEE Robot Autom Lett 2023; 8: 6531–8. 10.1109/LRA.2023.3308335

[16] Dong Y, Wang L, Zhang Z et al. Endoscope-assisted magnetic helical micromachine delivery for biofilm eradication in tympanostomy tube. Sci Adv 2022; 8: eabq8573. 10.1126/sciadv.abq8573 36206344 PMC9544342

[17] Deng B, Chen L, Wei D et al. Pulse-driven robot: motion via solitary waves. Sci Adv 2020; 6: eaaz1166. 10.1126/sciadv.aaz1166 32494671 PMC7195187

[18] Son D, Ugurlu MC, Sitti M. Permanent magnet array–driven navigation of wireless millirobots inside soft tissues. Sci Adv 2021; 7: eabi8932. 10.1126/sciadv.abi8932 34669466 PMC8528412

[19] Chen X, Goodnight D, Gao Z et al. Scaling up nanoscale water-driven energy conversion into evaporation-driven engines and generators. Nat Commun 2015; 6: 7346. 26079632 10.1038/ncomms8346 PMC4490384

[20] Fusi G, Del Giudice D, Skarsetz O et al. Autonomous soft robots empowered by chemical reaction networks. Adv Mater 2023; 35: 2209870. 10.1002/adma.202209870 36420882

[21] Wang H, Gao J, Xu C et al. Light-driven biomimetic nanomotors for enhanced photothermal therapy. Small 2024; 20: 2306208. 10.1002/smll.202306208 37670543

[22] Ahmed S, Wang W, Bai L et al. Density and shape effects in the acoustic propulsion of bimetallic nanorod motors. ACS Nano 2016; 10: 4763–9. 10.1021/acsnano.6b01344 26991933

[23] Gu G, Zhang N, Xu H et al. A soft neuroprosthetic hand providing simultaneous myoelectric control and tactile feedback. Nat Biomed Eng 2021; 5: 589–98. 10.1038/s41551-021-00767-0 34400808

[24] Polygerinos P, Correll N, Morin SA et al. Soft robotics: review of fluid-driven intrinsically soft devices; manufacturing, sensing, control, and applications in human-robot interaction. Adv Eng Mater 2017; 19: 100016. 10.1002/adem.201700016

[25] Yang D, Feng M, Sun J et al. Soft multifunctional bistable fabric mechanism for electronics-free autonomous robots. Sci Adv 2025; 11: eads8734. 10.1126/sciadv.ads8734 39888988 PMC11784860

[26] Zhang N, Ren J, Dong Y et al. Soft robotic hand with tactile palm-finger coordination. Nat Commun 2025; 16: 2395. 10.1038/s41467-025-57741-6 40064944 PMC11894155

[27] Yang X, Zhang N, Huang X et al. Multidirectional bending soft pneumatic actuator with fishbone-like strain-limiting layer for dexterous manipulation. IEEE Robot Autom Lett 2024; 9: 3815–22. 10.1109/LRA.2024.3369475

[28] Kalmar M, Boese A, Landes R et al. Injection and infusion technology disruption for use in MRI. Med Dev 2019; 12: 469–78. 10.2147/MDER.S216758 PMC6888236 31819677

[29] Fu S, Dong S, Shen H et al. Multifunctional magnetic catheter robot with triaxial force sensing capability for minimally invasive surgery. Research 2025; 8: 0681. 10.34133/research.0681 40276100 PMC12018763

[30] Shah A, Aran S. A review of magnetic resonance (mr) safety: the essentials to patient safety. Cureus 2023; 15: e47345. 38021512 10.7759/cureus.47345 PMC10657250

[31] Ceron S, Gardi G, Petersen K et al. Programmable self-organization of heterogeneous microrobot collectives. Proc Natl Acad Sci USA 2023; 120: e2221913120. 10.1073/pnas.2221913120 37276400 PMC10268276

[32] Yan Y, Wang T, Zhang R et al. Magnetically assisted soft milli-tools for occluded lumen morphology detection. Sci Adv 2023; 9: eadi3979. 10.1126/sciadv.adi3979 37585531 PMC10431716

[33] Dong X, Luo X, Zhao H et al. Recent advances in biomimetic soft robotics: fabrication approaches, driven strategies and applications. Soft Matter 2022; 18: 7699–734. 10.1039/D2SM01067D 36205123

[34] Haouas W, Gauthier M, Rabenorosoa K. Miniaturized soft robotics: recent advances and futures opportunities. Curr Robot Rep 2024; 5: 15–27. 10.1007/s43154-024-00109-3

[35] Chi Y, Zhao Y, Hong Y et al. A perspective on miniature soft robotics: actuation, fabrication, control, and applications. Adv Intell Syst 2023; 6: 20063.

[36] Purcell EM. Life at low Reynolds number. Am J Phys 1977; 45: 3–11. 10.1119/1.10903

[37] Fan D, Yuan X, Wu W et al. Self-shrinking soft demoulding for complex high-aspect-ratio microchannels. Nat Commun 2022; 13: 32859. 10.1038/s41467-022-32859-z PMC9424246 36038593

[38] Duraivel S, Laurent D, Rajon DA et al. A silicone-based support material eliminates interfacial instabilities in 3D silicone printing. Science 2023; 379: 1248–52. 10.1126/science.ade4441 36952407

[39] Li J, Cao J, Bian R et al. Multimaterial cryogenic printing of three-dimensional soft hydrogel machines. Nat Commun 2025; 16: 185. 10.1038/s41467-024-55323-6 39747822 PMC11695866

[40] Ji M, Li Q, Cho IH et al. Rapid design and analysis of microtube pneumatic actuators using line-segment and multi-segment Euler-Bernoulli beam models. Micromachines 2019; 10: 780. 10.3390/mi10110780 31739512 PMC6915588

[41] Jones TJ, Jambon-Puillet E, Marthelot J et al. Bubble casting soft robotics. Nature 2021; 599: 229–33. 10.1038/s41586-021-04029-6 34759362

[42] Becker K, Teeple C, Charles N et al. Active entanglement enables stochastic, topological grasping. Proc Natl Acad Sci USA 2022; 119: e2209819119. 10.1073/pnas.2209819119 36215466 PMC9586297

[43] Guo X, Tang W, Qin K et al. Powerful UAV manipulation via bioinspired self-adaptive soft self-contained gripper. Sci Adv 2024; 10: eadn6642. 10.1126/sciadv.adn6642 38718123 PMC11078182

[44] Bretherton FP. The motion of long bubbles in tubes. J Fluid Mech 1961; 10: 166–88. 10.1017/S0022112061000160

[45] Tsai TM, Miksis MJ. Dynamics of a drop in a constricted capillary tube. J Fluid Mech 1994; 274: 197–217. 10.1017/S0022112094002090

[46] Olbricht WL, Kung DM. The deformation and breakup of liquid drops in low Reynolds number flow through a capillary. Phys Fluids A 1992; 4: 1347–54. 10.1063/1.858412

[47] Jang Y, Nabae H, Suzumori K. Effects of surface roughness on direct plasma bonding between silicone rubbers fabricated with 3D-printed molds. ACS Omega 2022; 7: 45004–13. 10.1021/acsomega.2c05308 36530245 PMC9753519

