# Supplementary material for: Millimeter-scale fluid-driven soft robots
Source: Natl Sci Rev. 2025 Sep 29;12(11):nwaf413. doi: 10.1093/nsr/nwaf413 (PMC12598632; doi:10.1093/nsr/nwaf413)
Supplement: nwaf413_Supplemental_Files [file nwaf413_supplemental_files.zip › SupplementaryMaterial-Millimeter-scale fluid-driven soft robots For NSR-2025-690.docx]

Title:

Millimeter-scale fluid-driven soft robots

**Authors:**

Rong Bian1, Ninbin Zhang1, Xinyu Yang1, Jinhao Li1, Dezhi Yang1, Jieji Ren1, Jiang Zou1,2, Guoying Gu1,2*

**Affiliations:**

1 Robotics Institute and State Key Laboratory of Mechanical System and Vibration, School of Mechanical Engineering, Shanghai Jiao Tong University; Shanghai, 200240, China.

2 Meta Robotics Institute, Shanghai Jiao Tong University; Shanghai, 200240, China.

* Corresponding author. Email: Guoying Gu (guguoying@sjtu.edu.cn)

**Supplementary Text**

**Young-Laplace formula**

The pressure difference between the silicone liquid and the air bubble, due to the curved liquid surface, can be described by the Young-Laplace equation , where is the surface tension coefficient, and and are the principal curvatures of the curved liquid surface. The pressure inside the silicone liquid at a point is given by:

(S1)

where is the reference pressure (atmospheric pressure). Neglecting higher-order small quantities, the pressure difference between point A and point B **(Figure 2c)** is:

(S1.1)

**Navier-Stokes equation**

Silicone liquid is an incompressible fluid with high viscosity and low flow velocity. The Reynolds number for the flow of liquid in the tubular is very small, which is indicative of laminar flow. We assume that the silicone liquid has a constant density, is incompressible, and has a constant viscosity. The thickness of silicone layer on point A, , is on the millimeter scale, with surface forces acting on the liquid being much greater than gravitational forces, and the flow velocity is predominantly in the z-direction **(Figure 2c)**. Therefore the Navier-Stokes equation is

(S2)

Solving equation (S1) and equation (S2) and neglecting higher-order small quantities, we obtain the relationship between the flow velocity of the silicone liquid and its curvature:

(S3)

The boundary condition is and . The flow can be solved

(S4)

where replaces for concise formation **(Figure S1**). The rheological properties of silicone, , presents the confrontation between surface tension and viscous force. represents the unevenness of the interface.

Taking two surfaces, \#1 and \#2, separated by a distance , over a time interval, the volume of liquid flowing into \#1 is set with and that out of \#2 is set with . The liquid level height rises from to . Establish the equation of mass conservation:

(S5)

so

(S6)

The volume of liquid flow can be represented , we get

(S7)

Combined equation (S6) with equation (S7), we get

(S8)

Equation (S8) delineates the dynamic process whereby an initial perturbation incites a pressure gradient, which in turn induces a variation in the height of the liquid film. The left side of Equation (S8) accounts for the variation in the liquid film thickness, while the right side captures the pressure gradient resulting from surface tension forces. The equation (S8) has no analytical solution except on point A where ,. Supposing , is a positive dimensionless constant independent of size. The silicone height at point is

(S9)

where is the initial height of the silicone layer in point , and is the silicone viscosity. The height correlates positively with the surface tension coefficient and , and inversely with and . The index is 0.5 in theory if the thickness is assumed as constant. Considering that the viscosity of silicone grows during the instability, the index is smaller than the theoretical value and can be determined as based on **Figure 2d**. The .

Based on equation (S9), define the timespan for hill height to increase from to as , we can calculate :

(S10)

with .

**Theoretical minimal diameter by mini-BC**

The minimal diameter can be manufactured in the modified silicone with viscosity 100 and curing time 10 . Given that the coefficient of surface tension of common silicone is about , the theoretical minimal diameter is

(S11)

**Explanation of the fitting parameters**

In **Figure 2d**, parameters, and in equation [2], are determined by the nonlinear least squares method. A two-parameter model is fitted to the experimental traces using MATLAB’s trust-region-reflective algorithm, which minimizes the sum of squared residuals. Both and are dimensionless correction factors and carry no direct physical interpretation.

In **Figure 2e**, the plotted boundary represents the decision function of a binary classifier. A linear support-vector machine (SVM, MATLAB implementation) is trained on labelled data, and its slope is extracted directly from the resulting hyperplane.

**Mold preparation**

For soft actuators with a linear geometry, we choose the transparent tetrafluoroethylene pipe (PTFE tube, 3M Company) as the mold. Its smooth inner surface (Ra50 nm) enables high-quality outer surface of actuators. Besides, thanks to its transparent material, the internal condition of silicone liquid and bubble can be easily observed. For other nonlinear-shaped actuators, the mold can be created by 3D printing (X1 Carbon, Bambu Lab Inc.). To ensure the accuracy and smoothness of the contact surface between the mold and the silicone, we recommend that the mold-silicone contact surface be oriented upward in the printing setup. Before use, the mold should be pre-coated with a layer of surface treatment agent (Loctite 770 Surface Primer) to prevent silicone poisoning.

**The robot’s wall-thickness uniformity**

Wall-thickness uniformity is quantified by measuring the maximum and minimum on transverse cross-sections along the axial axis. Three representative actuators (D=1mm, 2.5mm, 4mm) are sectioned every 5 mm. The optical metrology (ImageJ, version 1.54f, National Institutes of Health, USA) records and versus axial position . Across all sizes, thickness variation remains within 5%, confirming fabrication uniformity.

To guarantee uniform wall thickness, three process parameters should be strictly controlled: (1) a constant bubble-propagation rate is imposed with a syringe pump instead of manual actuation; (2) the mold surface in contact with silicone is cleaned and kept dust-free immediately before casting; (3) the mold is cured on an actively vibration-isolated platform.

**Relationship between pressure and curvature**

In the absence of external contact, the milli-hooker’s curved segment adopts a perfectly circular arc of fixed length , yielding the geometric relation . The uniform curvature κ is determined solely by actuation pressure and is measured optically (**Figure S5**).

**Motion repeatability**

For the cycle test, a 1000-time cyclic load (0 kPa-25 kPa, 6 sec/cycle) is applied to the milli-actuator (**Figure S6**). Across all experimental runs, the actuator’s bending behavior exhibited no visible change. Neither failure nor any discernible performance degradation was observed.

**Advantages of Bronchoscopy Technologies**

**Table S4** summarizes key characteristics of state-of-the-art commercial bronchoscopes, magnetically actuated soft robots, and our work. Our milli-SRs exhibit a markedly lower effective elastic modulus (0.1-10MPa) than conventional bronchoscopes (500-1000MPa), minimizing bleeding risk and patient discomfort. Relative to magnetically driven counterparts, fluidic actuation is accomplished with only one or two handheld syringes, eliminating the need for large electromagnetic coils and robotic manipulators and thus affording superior portability and clinical readiness.

**Force quantification**

We test the milli-gripper (**Figure 3h**) and the contractile coiling milli-actuator (**Figure 3c**). Force calibration employs a precision sensor (FUTEK LSB200, 500mN range). The milli-gripper retains an ant until 220 mN of axial pull is applied (**Figure S8**a-b), whereas the coiling milli-actuator sustains 400 mN before complete uncoiling (**Figure S8**c-d).

**Comparative Benchmarking of Millimeter-Scale Fabrication Techniques**

Mini-bubble casting is benchmarked against injection-induced self-folding and silicone 3D printing across size scalability, surface roughness, functional performance, and structural complexity (**Table S6**). Relative to injection-induced self-folding, it enables seamless fabrication across multiple length scales; relative to 3D printing, it yields actuators with an order-of-magnitude reduction in surface roughness, substantially extending operational lifespan.

**Table S1. The suggested additives and their dosages to modify the stability condition.** With the suggested dosages, the enhanced silicone liquid presents its stability condition above the threshold. For platinum-cure silicones, the additives are usually added to part A. For tin-cure silicone rubber (Mold Max 10), the additives are usually added to part B.

|  |  | Outer diameter of milli-SRs | Thickner | Thickner dosage  (weight of part A) | Accelerator | Accelerator dosage  (weight of part A) |
| --- | --- | --- | --- | --- | --- | --- |
| **platinum cure** | Dragon Skin 10 | 3mm | THI-VEX | 0.50% | Plat-Cat | 1.00% |
|  |  | 2mm |  | 1.50% |  | 4% |
|  |  | 1mm |  | 4% |  | 8% |
|  | Dragon Skin 30 | 3mm | THI-VEX | 0.50% | Plat-Cat | 0.50% |
|  |  | 2mm |  | 1.50% |  | 2% |
|  |  | 1mm |  | 4% |  | 5% |
|  | Ecoflex 30 | 3mm | THI-VEX | 3% | Plat-Cat | 2% |
|  |  | 2mm |  | 4% |  | 3% |
|  |  | 1mm |  | 4.50% |  | 4% |
|  | RTV 615 | 3mm | Fumed Silica | 1% | Plat-Cat | 1% |
|  |  | 2mm |  | 1.50% |  | 2% |
|  |  | 1mm |  | 2% |  | 3% |
|  | Mold Star 16 | 3mm | THI-VEX | 0.50% | Plat-Cat | 1% |
|  |  | 2mm |  | 1.50% |  | 1.5% |
|  |  | 1mm |  | 4% |  | 2% |
|  |  |  |  | Thickner dosage  (weight of part B) |  | Thickner dosage  (weight of part B) |
| **Tin cur** | Mold Max 10 | 3mm | THI-VEX | 0.50% | Accel-T | 0.3% |
|  |  | 2mm |  | 1.50% |  | 1% |
|  |  | 1mm |  | 4% |  | 2% |
|  | Mold Max 40 | 3mm | THI-VEX | 0.25% | Accel-T | 0.5% |
|  |  | 2mm |  | 1% |  | 1.5% |
|  |  | 1mm |  | 2% |  | 3% |

**Table S2. Mini Bubble Casting duration.** Fabrication time: 10 min excluding cure; 20–70 min including cure.

|  | Step | Acquired time |
| --- | --- | --- |
| 1 | Prepare the silicone cartridge, pistons, and mixing nozzle | 1 min |
| 2 | Pour liquid silicone into the cartridge | 1 min |
| 3 | Add calculated amounts of accelerator and thickener, and mix | 3 min |
| 4 | Install pistons to seal the cartridge | 0.5 min |
| 5 | Assemble the setup into the cartridge gun | 0.5 min |
| 6 | Inject the silicone into the mold | 2 min |
| 7 | Introduce a bubble to form the internal void | 1 min |
| 8 | Waiting curing | 10-60min |
| 9 | Demold the milli-SRs | 1 min |
|  | Total operating timespan (excluding the silicone curing period) | 10 min |
|  | Total timespan (including the silicone curing period) | 20-70min |

**Table S3. List of Symbols**

| Parameter | Explanation |
| --- | --- |
| , , , | Height of silicone layer on point A, B, z, O |
| , , | Pressure in liquid silicone point (*r, , z*), A, and B |
|  | r-axis  the transformed r-axis  z-axis in cylindrical coordinates  *z*-component of velocity |
|  | The silicone viscosity |
|  | Surface tension coefficient between the silicone liquid and bubble |
| , , | Principal curvatures of silicone-bubble interface |
| , | Volume of liquid flowing in surface #1 /out of the surface #2 |
|  | Inner radius of the cylindrical mold |
|  | Elapsed time since bubble forming |
|  | Timespan for hill height to increase to its maximum, i.e. instability timespan |
|  | Stability condition |
|  | Curing time of liquid silicone |
|  | Threshold of stability condition |
| , | Positive dimensionless constant |

**Table S4. Advantages of bronchoscopy technologies**

|  | Commercial bronchoscopes [1] | Magnetically driven soft robots [2] | This work |
| --- | --- | --- | --- |
| Shell material | Polyurethane  500-1000MPa | Silicone  0.1-10MPa | Silicone  0.1-10MPa |
| Shell hardness | Hard | Soft | Soft |
| Drive source | Rope | Magnetic field | Pressure |
| Weight of driving system | Light | Heavy | Light |
| Degree of freedom | 1-2 | -- | 1-2 |
| Most distal site reached  (steerability) | 4th-order bronchus | 4th-order bronchus | 4th-order bronchus |
| Minimum outer diameter  (miniaturization) | 2.7 mm | 2 mm | 1 mm (1 void)  2.5 mm (3 voids) |
| Maximum bending angle  (operational flexibility) | ±180° | 81.47° | ±180° |

**Table S5. Comparative analysis of grippers**

|  | Metallic forceps  (Single-use biopsy forceps, Olympus) | Other fluid-driven soft gripper [3] | This work |
| --- | --- | --- | --- |
| Shell material | Mental | Silicone | Silicone |
| Young’s module | >1GPa | 0.1-10MPa | 0.1-10MPa |
| Shell hardness | Very hard | Soft | Soft |
| Risk of tissue damage | High | Low | Low |
| Dynamic response time | <0.5 s | ~1 s | ~0.5 s |
| Diameter of gripper | <3 mm | 10-30 mm | 1-2.5 mm |
| Minimal size of object grasped | 1 mm | 5 mm | ~1 mm |

**Table S6. Comparative Analysis of Multiple Fabrication Techniques**

|  | Injection-Induced Self-Folding [4] | 3D printing silicone [5] | This work  Mini bubble casting |
| --- | --- | --- | --- |
| Thickness of wall | 0.075 | 0.1 mm -0.5 mm | 0.05 mm - 1.5mm |
| Overall size | 0.1 mm–2 mm | 0.5 mm-15 mm | 1 mm – 1000 mm |
| Roughness | 10-50 nm | 0.5-1  μm | ~11 nm |
| Functional performance | Gripper, valve, mixer | Gripper | Gripper |
| Structural complexness | 3D | 3D | 3D |


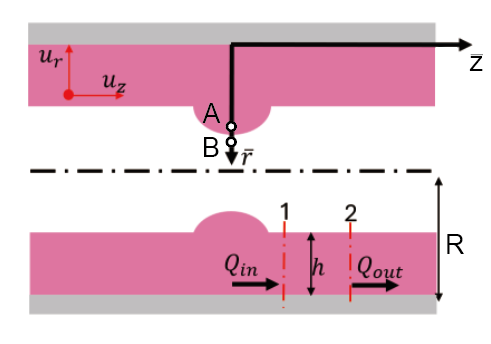


**Figure S1: Schematic diagram of silicone flow in new polar coordinate.** The new *r*-axis is from the mould to the center line (). The height is controlled by the volume of silicone flow on plane #1, , and silicone flow on plane #2, .


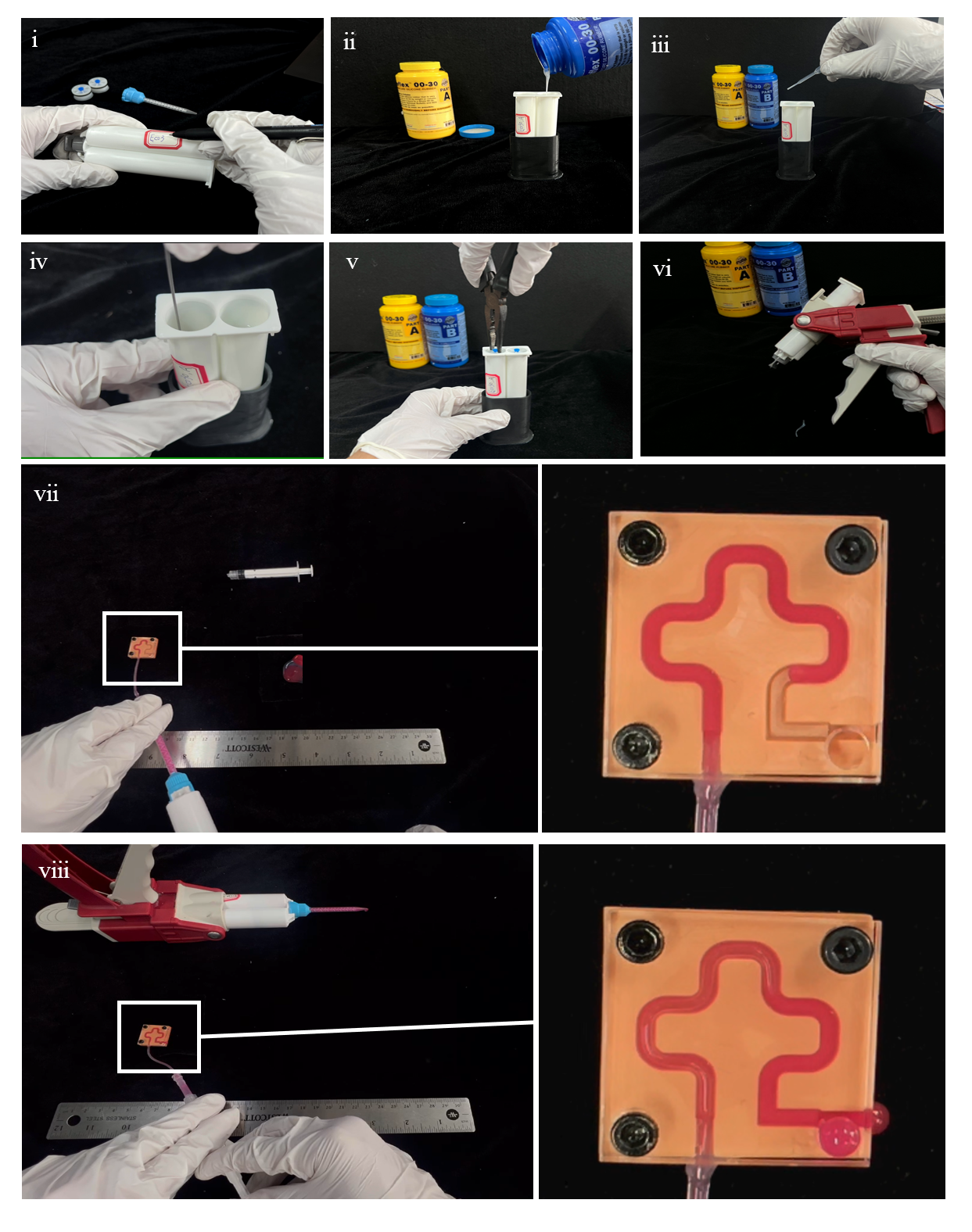


**Figure S2: Mini-BC process in the laboratory.** Prepare the silicone cartridge, pistons, and mixing nozzle. (i) Pour liquid silicone into the cartridge, (iii) add calculated amounts of accelerator and thickener, and mix thoroughly. (v) Install pistons to seal the cartridge and (vi) assemble the setup into the cartridge gun, (vii) inject the silicone into the mold, and (viii) introduce a bubble to form the internal void


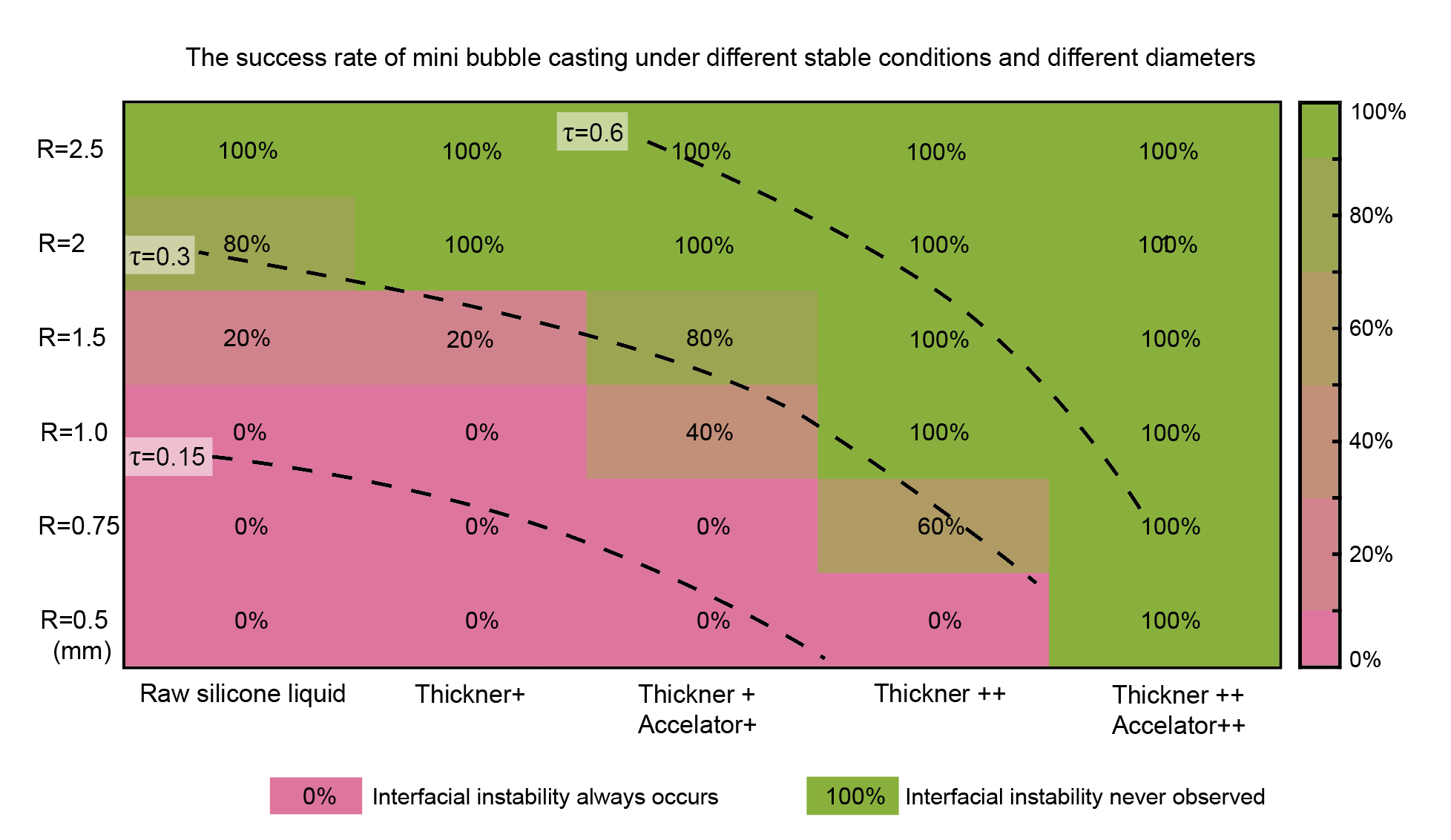


**Figure S3: The fabrication yield of mini bubble casting across various instability conditions and diameters.** For each (instability condition, diameter) pair, five independent trials have been performed using Dragon Skin 10 Fast, a trial is deemed successful when interfacial instability is suppressed.


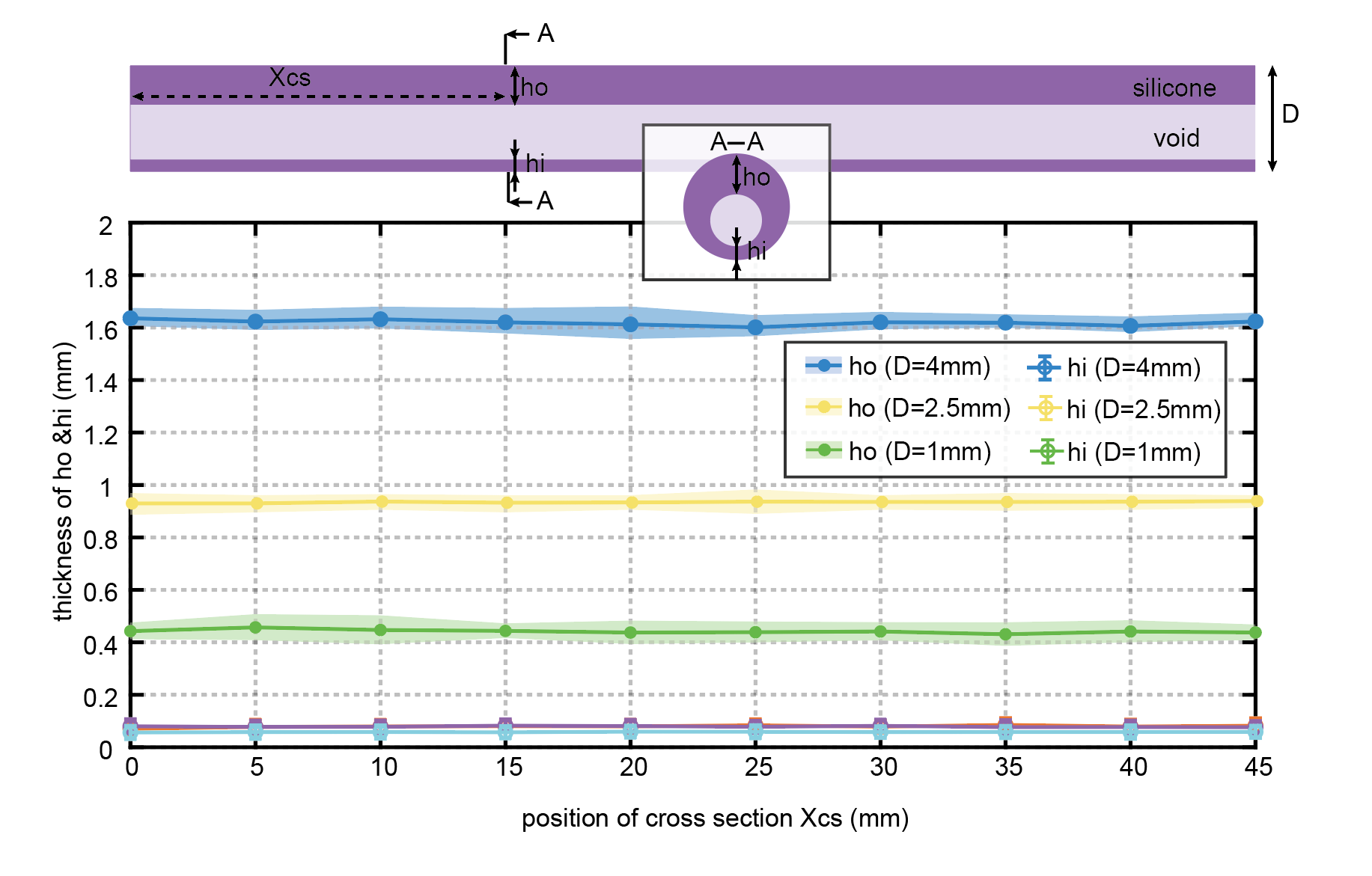


**Figure S4: The robot’s wall-thickness uniformity.** The maximum wall thickness, and the minimum value on each transverse section at different axial positions is evaluated every 5 mm along the axial direction for three representative actuators (D=1mm, 2.5mm, 4mm).

**
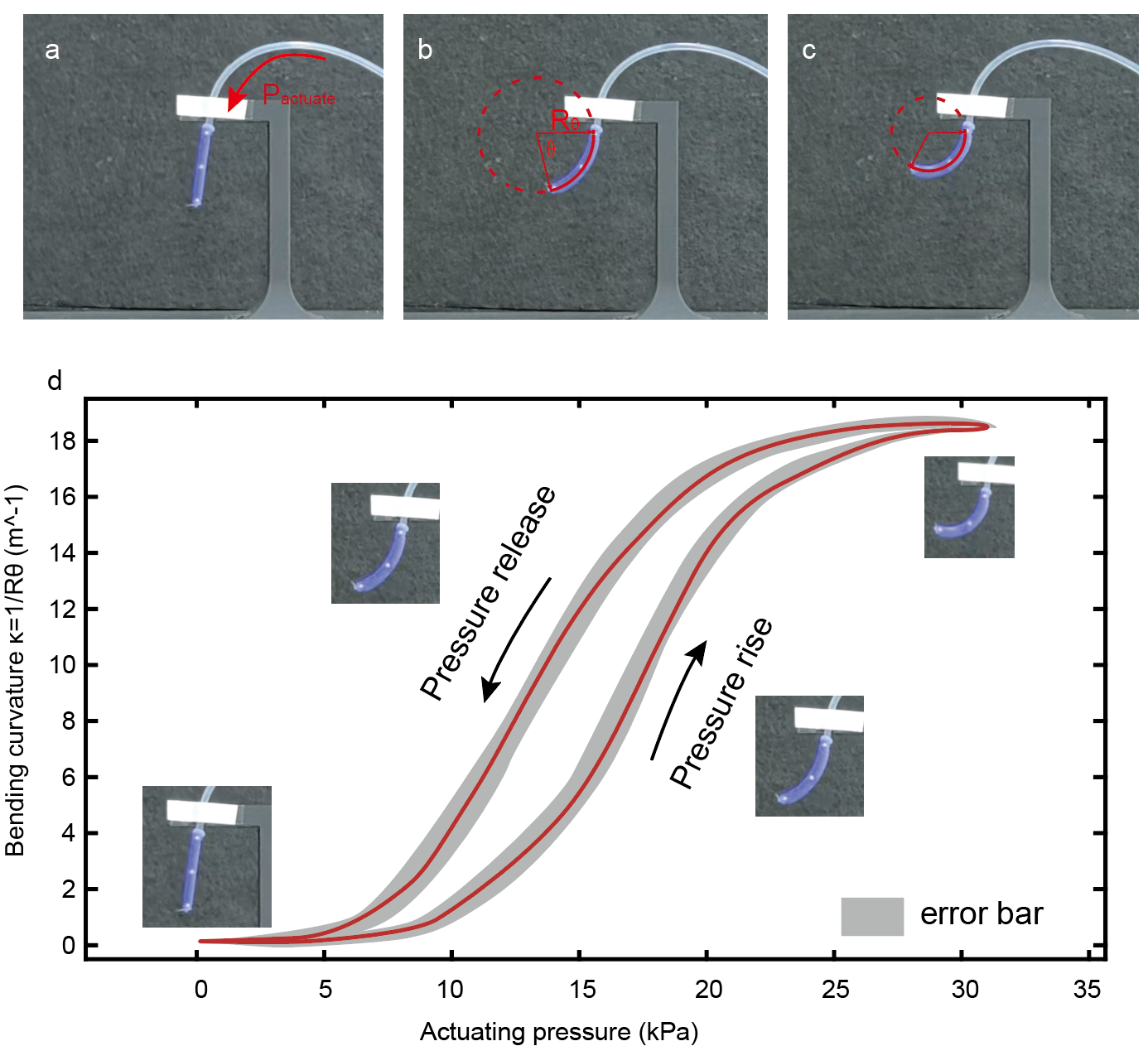
**

**Figure S5: The pressure-curvature relationship.** (a-c) The pressure rising stage. (d) The relationship between the actuating pressure and the curvature of the actuator; the grey area is the error bar calculated by three-cycle loading tests.


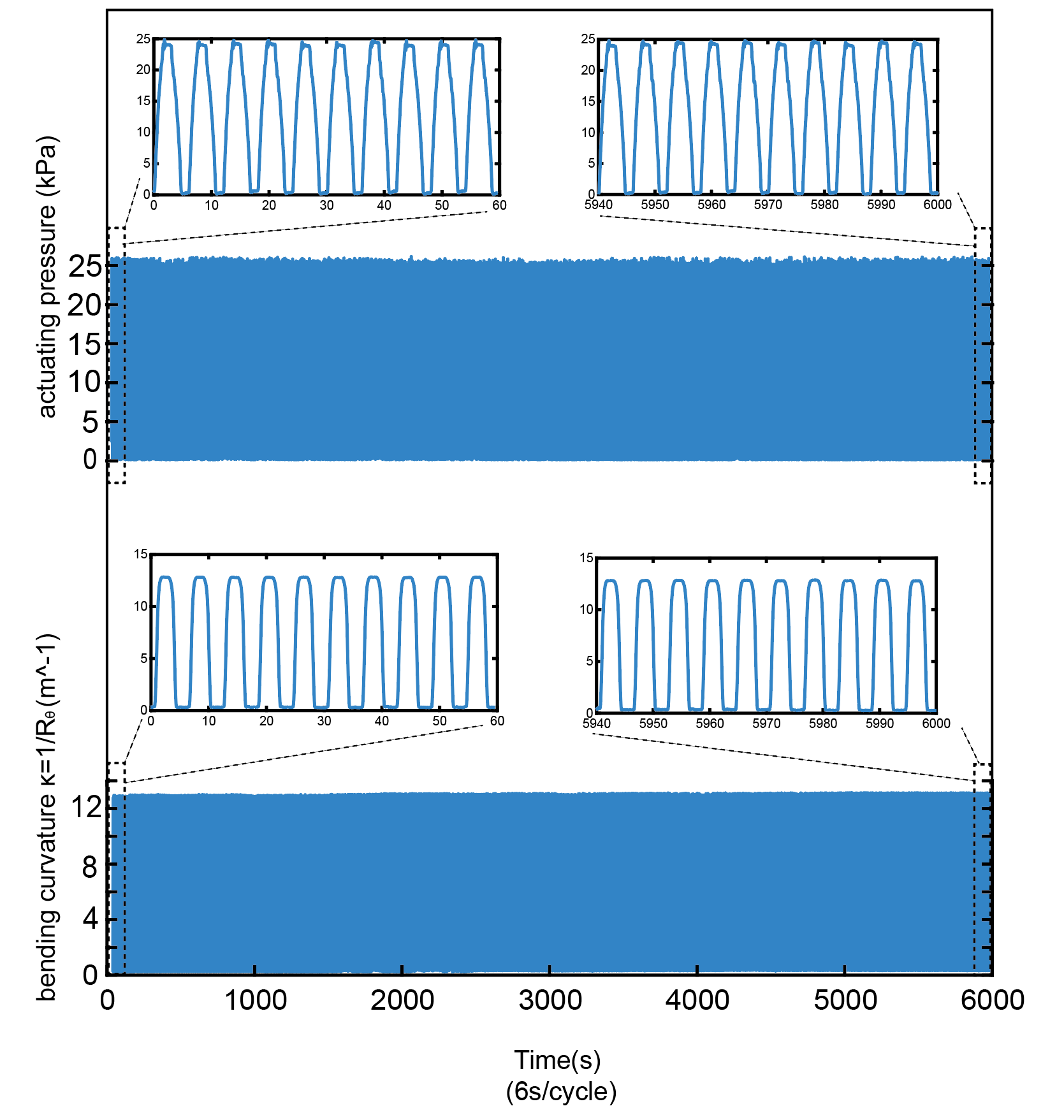


**Figure S6: The repeatable motion under 1000-time cyclic load.** In one cycle (cycle time = 6 sec), the actuator is subjected to pressure loading and unloading at 25 kPa, while its bending curvature is recorded.

###
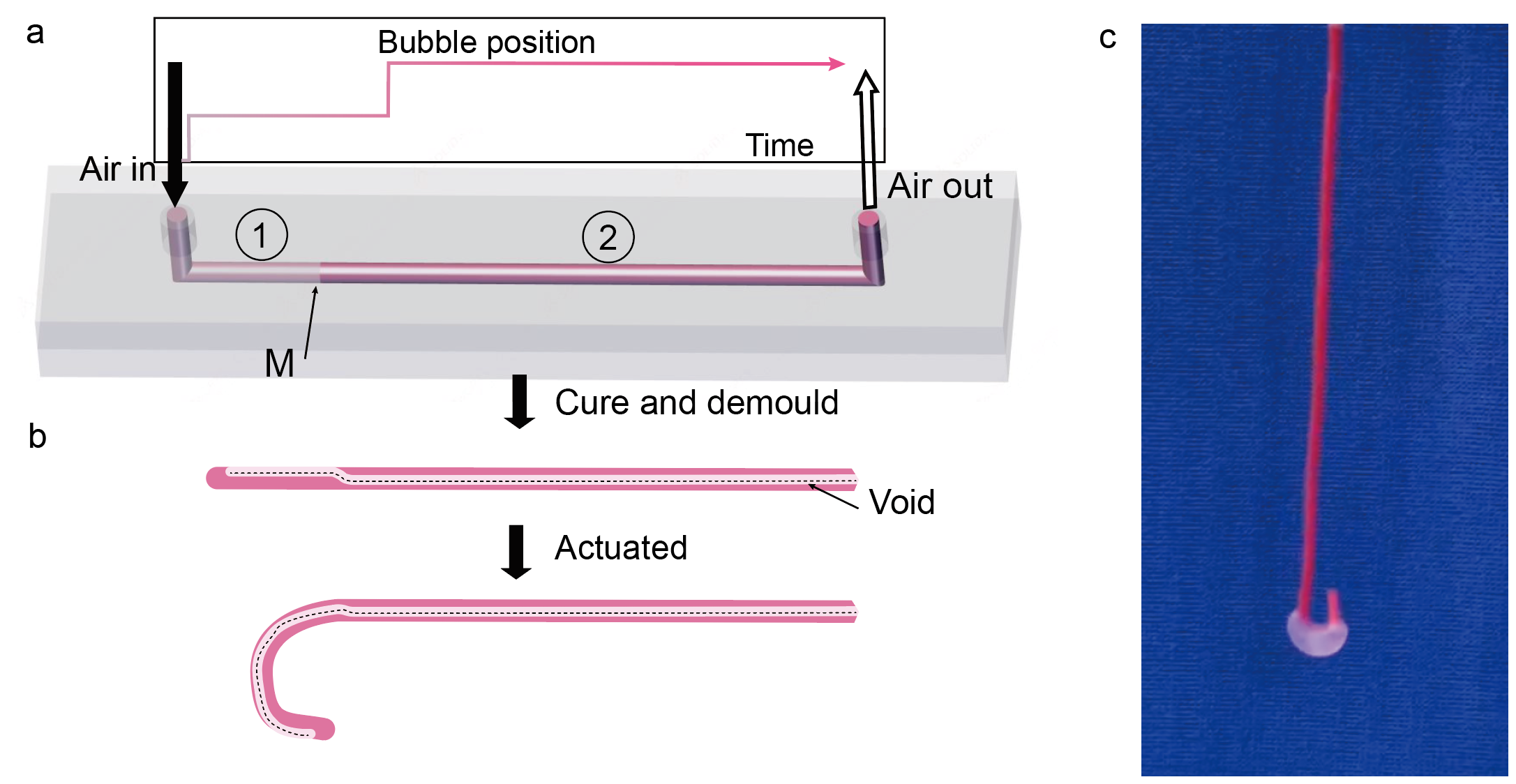


**Figure S7: Fabrication of the milli-hook actuator.** (a) The silicone is injected into the mould, followed by the introduction of a bubble at the designated location M. After a prescribed waiting period, pressured air is gradually increased to propel bubble toward the outlet. (b) Upon curing, the silicone actuator exhibits an internal void that varies axially. At the actuator’s left end, the void is positioned closer to the upper surface, while it is nearly centrally located in the right region. (c) The axial variation in the void’s position allows it to deform into a hook-like shape.


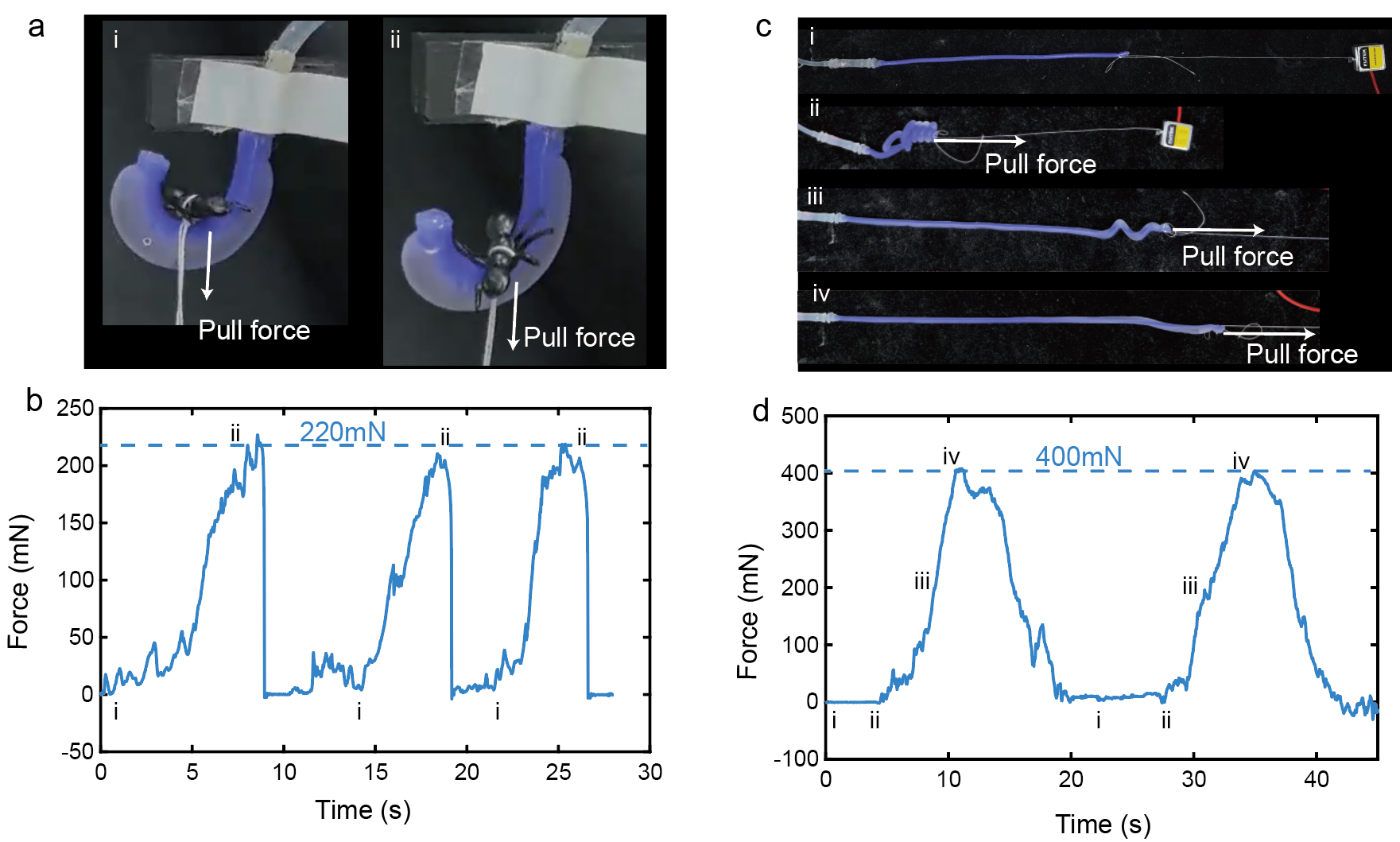


**Figure S8: Force quantification of the milli-gripper and the coiling actuators.** (a-b) Dislodgement of the ant from the milli-gripper requires a pulling force of ≈ 220 mN. (c-d) A tensile load is applied to the pressurized milli-actuator until the coil is fully extended.

### **
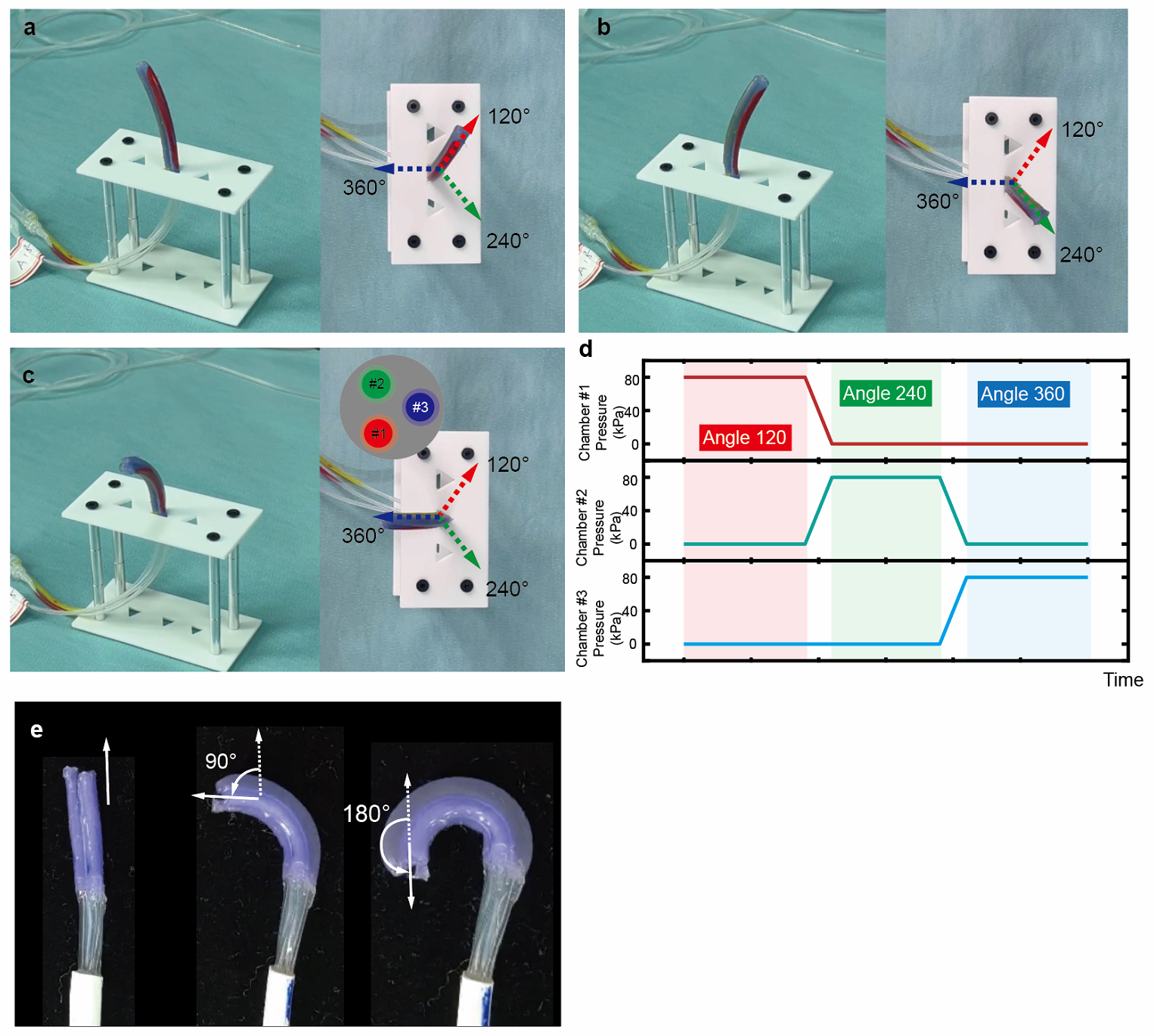
**

**Figure S9: Steerable distal tip of the soft bronchoscopy.** (a-c) Directional actuation is achieved by selective pressurization of the three circumferential voids. (d) Independent syringe pumps supply regulated air pressure to each void. (e) The tip attains a maximum bending angle > 180°.

*
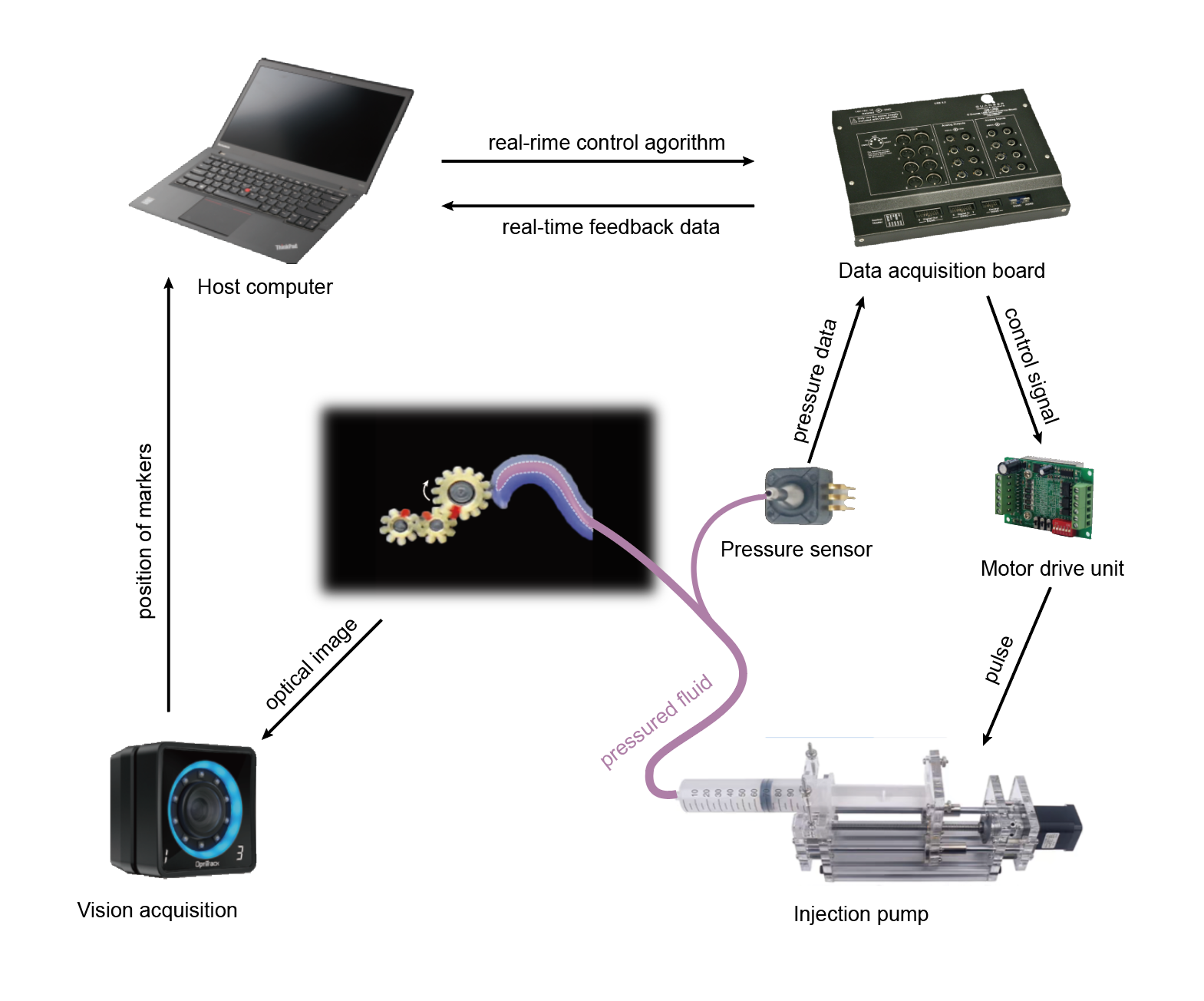
*

**Figure S10: The closed-loop control system of actuation pressure.** The pump’s drive unit regulates output while a pressure sensor provides real-time feedback. A data-acquisition board relays sensor data to the host computer, which also processes visual feedback for supervisory control.

Reference:

[1] Kristensen AE, Kurman JS, Hogarth DK, et al. Systematic Review and Cost-Consequence Analysis of Ambu aScope 5 Broncho Compared with Reusable Flexible Bronchoscopes: Insights from Two US University Hospitals and an Academic Institution. *Pharmacoecon Open* 2023; **7**: 665-67.

[2] Pittiglio G, Lloyd P, da Veiga T, et al. Patient-Specific Magnetic Catheters for Atraumatic Autonomous Endoscopy. *Soft Robot* 2022; **9**: 1120-1133.

[3] Zhang N, Ren J, Dong Y, et al. Soft robotic hand with tactile palm-finger coordination. *Nat Comm* 2025; **16**: 2395.

[4]Ranzani Y, Russo S, Bartlett NW, et al. Increasing the Dimensionality of Soft Microstructures through Injection‐Induced Self‐Folding. *Adv Mater;* 2018;**30**: 1802739 .

[5]Qing H, Chi Y, Hong Y, et al. Fully 3D‐Printed Miniature Soft Hydraulic Actuators with Shape Memory Effect for Morphing and Manipulation. *Adv Mater* 2024*;* **36**: 2402517.
